# Evolution of bone densitometry parameters and risk of fracture in coeliac disease: a 10-year perspective

**DOI:** 10.1007/s11739-023-03307-7

**Published:** 2023-06-03

**Authors:** Francesco Tovoli, Dante Pio Pallotta, Alice Giamperoli, Guido Zavatta, Kinga Skoracka, Alberto Raiteri, Chiara Faggiano, Iwona Krela-Kaźmierczak, Alessandro Granito

**Affiliations:** 1 Department of Medical and Surgical Sciences, University of Bologna, Bologna, Italy; 2 Division of Internal Medicine, Hepatobiliary and Immunoallergic Diseases, IRCCS Azienda Ospedaliero-Universitaria di Bologna, Bologna, Italy; 3 Division of Endocrinology and Diabetes Prevention and Care, IRCCS Azienda Ospedaliero-Universitaria di Bologna, Bologna, Italy; 4 Doctoral School, Poznan University of Medical Sciences, Poznan, Poland; 5 Department of Gastroenterology, Dietetics and Internal Diseases, Poznan University of Medical Sciences, Heliodor Swiecicki Hospital, Poznan, Poland

**Keywords:** Coeliac disease, Osteoporosis, Bone, Dual-energy X-ray absorptiometry, Fractures, Gluten, Gluten-free diet

## Abstract

**Background:**

Metabolic bone disease is frequently found in patients with coeliac disease (CD). Despite its high prevalence, international guidelines are partially discordant about its management due to the lack of long-term data.

**Methods:**

We retrospectively evaluated a large dataset of prospectively collected data of CD patients assessing the variation of DXA parameters and estimated fracture risk according to the FRAX^®^ score in a 10-year follow-up. Incident fractures are reported, and the predictive ability of the FRAX^®^ score is verified.

**Results:**

We identified 107 patients with low bone density (BMD) at the diagnosis of CD and a 10-year follow-up. After improving at the first follow-up, T-scores slowly reduced over time but with no clinically relevant differences between the first and last examination (lumbar spine: from − 2.07 to − 2.07, *p* = 1.000; femoral neck: from − 1.37 to − 1.55, *p* = 0.006). Patients with osteoporosis at the index measurement had more marked fluctuations than those with osteopenia; the latter group also showed minimal modifications of the FRAX^®^ score over time. Six incident major fragility fractures occurred, with a good predictive ability of the FRAX^®^ (AUC 0.826).

**Conclusion:**

Adult CD patients with osteopenia and no risk factors had substantially stable DXA parameters and fracture risk during a 10-year follow-up. A dilated interval between follow-up DXA for these patients could be considered to reduce diagnosis-related time and costs, maintaining a 2-year interval for patients with osteoporosis or risk factors.

**Supplementary Information:**

The online version contains supplementary material available at 10.1007/s11739-023-03307-7.

## Introduction

Osteoporosis can be found in 26–40% of patients at the diagnosis of coeliac disease (CD) [1–3], resulting in an excess risk of fractures of 320–480/100000 person-years in adults [4–7]. Treatment with a gluten-free diet (GFD) improves bone mineral density after 1–2 years of adherence [8–11], but longer term data are lacking. The effects of the GFD on reducing the risk of fragility fractures are still debated, as population-based studies found a similar incidence ratio for fractures before and after CD diagnosis [6]. Thus, the protective role of GFD on subsequent fracture risk may not be universal [12]

As a consequence of these uncertainties and lack of long-term data, the main international guidelines provide partially discordant recommendations. The American College of Gastroenterology (ACG) guidelines recommend a dual-energy X-ray absorptiometry (DXA) at the diagnosis of CD and every 2–3 years [12]. The European Society for the Study of Coeliac Disease (ESsCD) also recommends DXA at the diagnosis of CD, but limits follow-up DXAs to patients with abnormal index values [13]. Conversely, the British Society of Gastroenterology (BSG) recommend a DXA evaluation in patients with risk factors for osteoporosis or if > 55 year-old, repeating the investigation only in patients with low bone density on index measurement [14]. Finally, the National Institute for Health and Care Excellence (NICE) recommend a preliminary risk assessment through clinical scores, prescribing DXA only over certain risk thresholds and monitoring only patients receiving treatments [15].

Answering the call for research favoring an optimized management of bone disease in CD [13] and trying to provide data helping in standardizing the current recommendations, we designed a study gathering information starting from the index DXA and extending for 10 years.

## Methods

### Clinical setting

We retrospectively analyzed the medical records of patients consecutively diagnosed with CD in our outpatient clinic (IRCCS Azienda Ospedaliero-Universitaria di Bologna, Bologna, Italy) between January 2004 and December 2020. The database was locked in February 2022. The target population was represented by CD patients who had low BMD at the index DXA examination and received regular follow-up DXAs every 2–3 years as part of everyday clinical practice, according to the European recommendations.

Overtime variations of the main DXA parameters and risk of fracture estimated according to the Fracture Risk Assessment Tool (FRAX^®^) score were the main aim. The secondary aim was to assess the actual rate of major fragility fractures and the predictive ability of the FRAX score in identifying patients who had incident fractures. FRAX^®^ is a tool based on individual patient models that integrate the risks associated with both clinical risk factors and bone mineral density [16]. The output is a 10-year probability of a major osteoporotic fracture. The calculated probability can be used to guide treatment strategies. A > 20% probability of major osteoporotic fractures is widely recognized as a threshold to define high-risk patients which should be considered for antiresorptive treatments. More recently, a 10% probability threshold has been proposed to discriminate low-risk and moderate-risk (10–20% probability) patients [17]. This proposal reflected the fact that more osteoporotic fractures occur in the moderate-risk group than the high-risk group as an absolute value (because there are more individuals in the moderate-risk group), even though the individual risk of fracture remains higher in the high-risk group [18]. Also, according to a recent consensus [19], a 10% risk in a relatively young population is high enough to consider pharmacological treatment in selected cases. On the contrary, some agencies are known to be reluctant to reimburse treatments on the basis of fracture probability at younger ages when the 10-year probability of a major osteoporotic fracture is less than 10% [19]. For these reasons, a 10% risk threshold was set as a cut-off for the subgroup analyses in this study, which included a relatively young population.

### Inclusion and exclusion criteria

Coeliac disease was diagnosed by serologic testing of coeliac-specific antibodies (anti- transglutaminase IgA, or IgG in patients with IgA deficiency) and confirmed by duodenal mucosal biopsies [12]. Classical (i.e., with signs and symptoms of malabsorption) and non-classical CD were defined according to the Oslo criteria [20]. We included patients with the following additional criteria: (1) availability of an index DXA, performed within 12 months from the diagnosis of CD; (2) low BMD, defined as abnormal T-score values (< − 1.0) at the index DXA; (3) a minimum 10-year follow-up after the diagnosis of CD; (4) follow-up DXAs regularly performed at 24–36 months intervals after the index examination.

Patients were excluded in case of: (1) index or follow-up DXAs performed outside of the timeframe; (2) incomplete medical records.

The following data were available for all patients: age at the diagnosis, sex, weight, height, family history of CD, clinical presentation (classical vs non-classical), adherence to the GFD (defined as no reported intentional or accidental gluten ingestion in the last 6 months, absence of CD‐related symptoms, and negative anti‐transglutaminase IgA antibodies) [21]. Medical records systematically included information about: family history for fragility fractures, personal history regarding smoke and alcohol habits, bone fractures, concurrent illnesses, and ongoing medications.

### Clinical, laboratory, and DXA evaluations

Clinical evaluations were scheduled according to the Italian Protocol for the Diagnosis and Follow-Up of Coeliac Disease (a first follow-up visit 6 months after the start of the GFD, then every 18–24 months) [22].

Blood tests were repeated before each evaluation, including blood cell count, ferritin, TSH, 25-OH vitamin D, total calcium, and anti-tissue transglutaminase IgA.

An index DXA exploring the lumbar spine and hip was prescribed at the diagnosis of CD. Differences between the bone mineral density of each patient and the young adult reference (T-score) or the same age and sex reference (Z-score) were expressed as standard deviations. If the T-score was below the normal range (i.e., < − 1.0) in at least one site, follow-up DXAs were prescribed every 24–36 months. On the contrary, no further DXAs were scheduled for patients with normal values unless new risk factors appeared [12, 13, 22]. Osteopenia (T-score between − 1.0 and − 2.5), osteoporosis (T-score ≤ − 2.5), and BMD below/within the expected range for age (Z-score ≤ or > 2.0, respectively) were defined according to the classification of the World Health Organization and the International Society for Clinical Densitometry (ISCD) [23, 24] DXA scans were performed and analyzed following manufacturer recommendations [25]. Patients were strongly suggested to perform follow-up DXAs with the same machine as the one used at their index examination to minimize the risk of non-comparable results. BMD values were expressed as either T-scores or Z-scores to allow BMD comparison over time. Of note, BMD values derived from different DXA machines are still well correlated [26].

### Risk of fracture

Fracture risk was retrospectively calculated at each timepoint using the Fracture Risk Assessment Tool (FRAX.^®^) for the Italian population (available at https://www.sheffield.ac.uk/FRAX/tool.aspx?lang=en) [16].

For patients younger than 40-year old, the calculator automatically inserted 40 years. Coeliac disease was considered a cause of secondary osteoporosis in calculating the FRAX score to maximize its predictive abilities [27].

### Fractures

During each clinical evaluation, patients were asked whether they had experienced a bone fracture. Medical records were also checked to reduce the risk of unreported events. According to the definitions of the World Health Organization, a fragility fracture was recorded if it resulted from mechanical forces that would not ordinarily result in a fracture. Fractures of the spine (clinical), hip, wrist, and humerus were considered major osteoporotic fractures (MOF). Asymptomatic vertebral fractures found at the X-ray examinations were considered previous fractures [23].

### Statistics

Categorical variables have been reported as frequencies (percentage). Continuous variables are expressed as mean and standard deviation (SD). Mixed ANOVA for repeated measures was used to assess the overtime variations of the DXA parameters and the possible influence of clinical factors. T- and Z-scores at each of the five timepoints were used as the within-subjects factor. At the same time, clinical variables (age, sex, osteoporosis at the diagnosis, menopause) were considered between-subjects factors. Mauchly’s Test was used to assess sphericity, and a Greenhouse–Geisser correction was applied in case of violation of sphericity. Post hoc analyses were performed with a Bonferroni adjustment. A *p* < 0.05 was considered statistically significant for all the analyses.

The diagnostic power for the detection of osteoporosis-related fracture of FRAX was estimated using the receiver operator characteristic (ROC) curve analysis.

### Ethics

This study was approved by the Institutional Review Board of the Bologna Authority S.Orsola-Malpighi Hospital (Protocol 243/2013/O/OssN) and performed according to the Declaration of Helsinki guidelines. Informed consent was obtained according to Institutional Review Board instructions.

### Patient and public involvement

Patients or the public were not involved in the design, or conduct, or reporting, or dissemination plans of our research.

## Results

### Study population

Among 1202 patients in our database, 1066 had an index DXA at the diagnosis of CD. Of them, 601 (56.4%) had normal results. The remaining 465 (43.4%) had a T-score < − 1.0 in at least one site and were followed up. Among them, 120 patients had a follow-up > 10 years. Thirteen patients, however, had missing or out-of-window data and were excluded. Thus, the final study population included 107 patients.

### Baseline characteristics

Most of patients were females (*n* = 88, 82.2%), among whom 35 were in menopause. The mean age was 43.4 years (SD 13.3), with 50.5% of patients being > 40-year-old. Thirty-four (31.8%) patients had a classical presentation of CD. The prevalence of factors favoring a condition of osteoporosis is reported in Table 1.

**Table 1:** Demographics and factors associated with bone mineral loss in the study population

Variable	
Age (years)	43.4 ± 13.3
Females	88 (82.2)
- *of whom, menopause*	*35 (39.8)*
Classical presentation of CD	34 (31.8)
Cigarette smoke	10 (9.3)
Systemic glucocorticoids	6 (5.6)
Inflammatory bowel disease	3 (2.8)
Chronic obstructive pulmonary disease	2 (1.9)
Autoimmune hepatitis	1 (0.9)
Previous fragility fractures	5 (4.7)
Spine	4 (3.7)
Wrist	1 (0.9)
Endocrine diseases	5 (4.7)
Type 1 diabetes mellitus	2 (1.9)
Hyperthyroidism	2 (1.9)
Panhypopithuitarism	1 (0.9)
Familiarity for major fragility fractures	4 (3.7)
Concurrent medications other than GC	4 (3.7)
Tamoxifene	3 (2.8)
Anticonvulsivants	1 (0.9)
Underweight	3 (2.8)
Alcohol consumption > 3 units/day	2 (1.9)
Primary biliary cirrhosis	2 (1.9)
Psoriariatric arthritis	1 (0.9)

Age is expressed ad mean and standard deviation. Prevalence is reported as frequency (percentage)

The mean T-scores were − 2.1 (SD 0.8) and − 1.4 (SD 0.7) at the lumbar spine and hip, respectively. According to their T-scores, 32 (29.9%) patients had osteoporosis, and 75 (70.1%) had osteopenia. Among the 32 patients with osteoporosis, 6 had T-scores < − 2.5 at both spine and hip, while 26 only at the spine.

In a multivariable model including age at the diagnosis (categorized as < 40 or ≥ 40 years), sex, body mass index, family history of CD, and clinical presentation (classical vs. non-classical), both age ≥ 40 years [odds ratio 5.11, 95% confidence interval 1.86–14.03, *p* = 0.002] and classical presentation [odds ratio 4.95, 95% confidence interval 1.87–13.17, *p* = 0.001] were independently associated with osteoporosis.

The mean Z-scores were − 1.6 (SD 0.7) and − 1.0 (SD 0.7) at the lumbar spine and total hip, respectively. Based on their Z-scores, 31 (29.0%) patients had an inadequate bone mineral density for age. In this case, the alterations were found in both sites in 5 patients and in a single site in 26 patients (spine *n* = 25, hip *n* = 1). Classical presentation was the only factor independently associated with BMD below the expected range for age according to the multivariable regression model [odds ratio 2.81, 95% confidence interval 1.17–6.84, *p* = 0.021].

The mean estimated 10-year risk of major fragility fractures was 4.0% according to the FRAX algorithm, with eight (7.5%) patients exceeding the 10% risk threshold.

After the baseline evaluation, Vitamin D and calcium were supplemented in 83 (77.6%) and 67 (62.6%) patients. Twenty-two (20.6%) patients were prescribed with bisphosphonates.

### Follow-up—changes in clinical data and evolution of DXA-based parameters

**Fig. 1:**
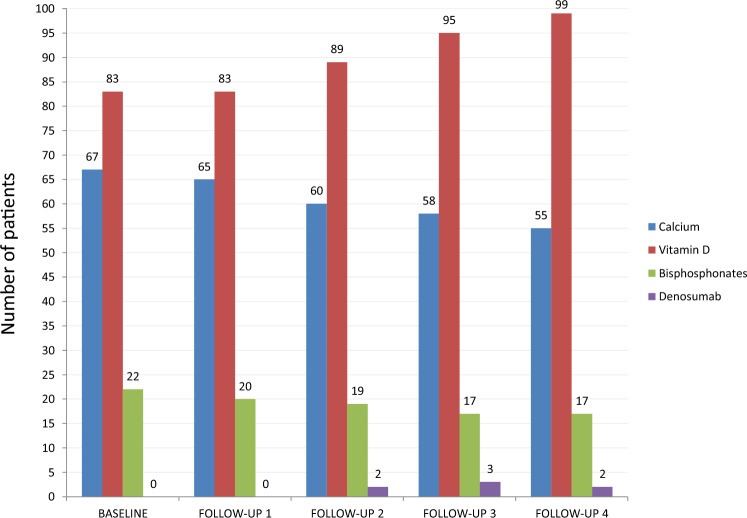
Supplements and drugs prescribed at the first evaluation and during the follow-up

The follow-up DXA were performed 2.4 (SD 0.5), 5.0 (SD 1.0), 7.4 (SD1.1), and 10.3 (SD 1.1) years after the first examination. During the follow-up, 18 women transitioned to menopause, with a medium age at menopause of 50.2 (SD 2.1) years. Additional risk factors for fracture appeared in six patients (in most cases, corticosteroids were started to treat concurrent conditions). Six (5.6%) patients had an incomplete adherence to the GFD. Prescription patterns of calcium, vitamin D, and antiresorptive drugs are reported in Fig. 1.

**Fig. 2:**
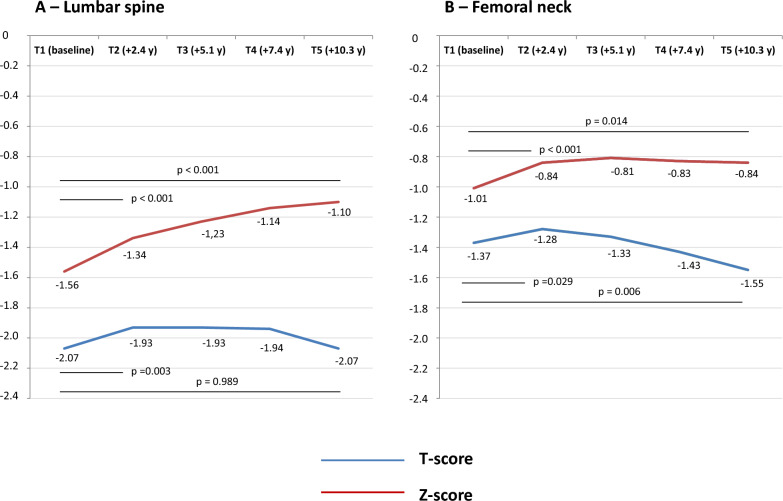
Evolutive changes of dual-energy X-ray absorptiometry parameters in the study population (*n* = 107)

From a descriptive point of view, T- and Z-scores at the lumbar spine and hip had an upward trend at the first follow-up, with different subsequent patterns according to the site (Fig. 2).

The mixed ANOVA for repeated measures confirmed variation of T-scores over time, both at lumbar spine [*F* (2.563, 271.687) = 4.929, *p* 0.001] and at the hip [*F* (2.763, 292.887) = 9.873, *p* < 0.001]. Post hoc analysis revealed that values at the lumbar spine improved from the baseline to the first follow-up (*p* = 0.002) and that there were no significant differences between baseline and the last follow-up (*p* = 1.000). Instead, the differences between the baseline and the first (*p* = 0.089) and last follow-up (*p* = 0.072) were borderline significant at the hip.

The ANOVA also confirmed Z-scores variation over time, both at lumbar spine [*F* (2.686, 265.906) = 20.038, *p* < 0.001] and at the hip [*F* (2.642, 280.037) = 6.091, *p* = 0.001]. The post hoc analysis at the lumbar spine showed that all follow-up values significantly increased (*p* < 0.001 in all cases). At the hip, the scores improved at the first follow-up (*p* = 0.002); this benefit remained steady, with a significant difference at the last follow-up (*p* = 0.006).

Finally, the mixed ANOVA model did not show significant interactions between variations in DXA parameters and sex (T-score lumbar spine *p* = ; T-score hip *p* = ; Z-score lumbar spine *p* = 0.938; Z-score hip *p* = 0.699), age > 40 years at the diagnosis (T-score lumbar spine *p* = 0.273; T-score hip *p* = 0.390; Z-score lumbar spine *p* = 0.508; Z-score hip *p* = 0.206), or menopause (T-score lumbar spine *p* = 0.878; T-score hip *p* = 0.812; Z-score lumbar spine *p* = 0.910; Z-score hip *p* = 0.924). With the strong limitation of the small number of non-adherent patients (*n* = 6), no statistically significant interactions were found for dietary adherence. Instead, DXA parameters variations were influenced by osteoporosis at the diagnosis (T-score lumbar spine *p* = 0.002; T-score hip *p* = 0.496; Z-score lumbar spine *p* < 0.001; Z-score hip *p* < 0.001).

**Fig. 3:**
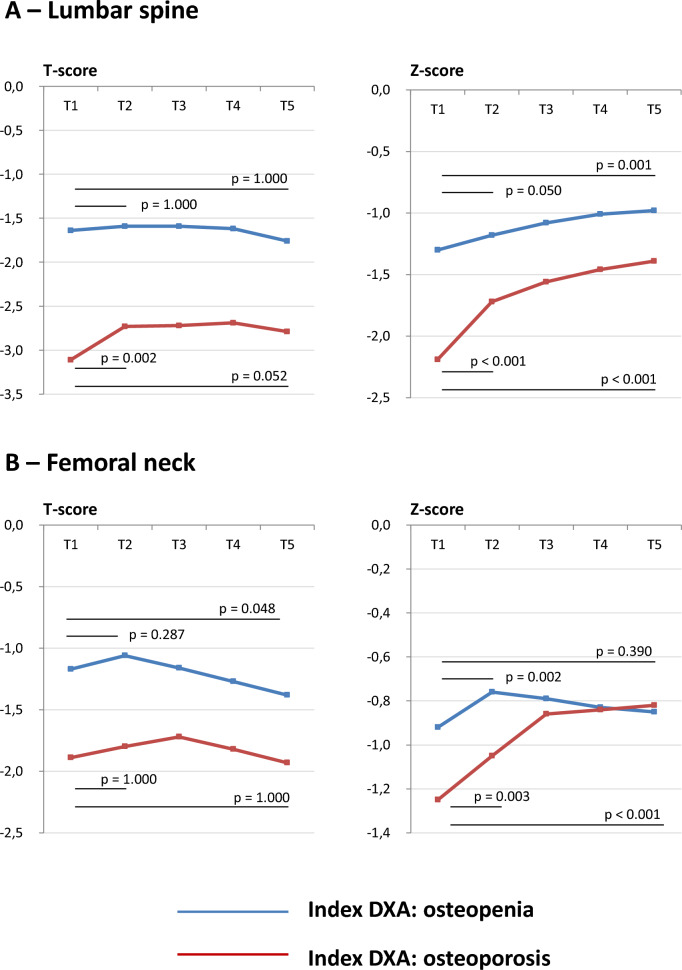
Evolutive changes of dual-energy X-ray absorptiometry parameters stratified according to the presence of osteoporosis (*n* = 32) or osteopenia (*n* = 75) in at least one site at the index examination

Consequently, separated subgroup analyses for patients with osteopenia vs. osteoporosis were performed. These analyses confirmed different trajectories of the DXA parameters. In particular, patients with osteoporosis had sharper increases in both T- and Z-scores; on the contrary, patients with osteopenia had relatively more stable values across the 10-year observation (Fig. 3).

### Follow-up—changes in the FRAX score

The evolution of the fracture risk estimated according to the FRAX algorithm (both reported as a continuous variable and categorized according to different thresholds) is reported in Table 2.

**Table 2:** Evolutive changes of the 10-year risk of major fragility fractures estimated according to the Fracture Risk Assessment Tool (FRAX^®^) in the study population (*n* = 107)

	Baseline (T1)	T2	T3	T4	T5	*p* (T1 vs. T5)
Mean risk	4.1	4.2	4.5	5.1	6.1	< 0.001
Median risk	2.7	2.8	3.1	3.5	4.2	< 0.001
< 10% risk	98 (91.6)	100 (93.5)	98 (91.6)	96 (89.7)	92 (86.0)	0.077
≥ 10% risk	9 (8.4)	7 (6.5)	9 (8.4)	11 (10.3)	15 (14.0)	

Mean risks were compared using a *t* test for repeated measures, median risks with Wilcoxon signed-rank tests, and the categorized values using the McNemar test

**Fig. 4:**
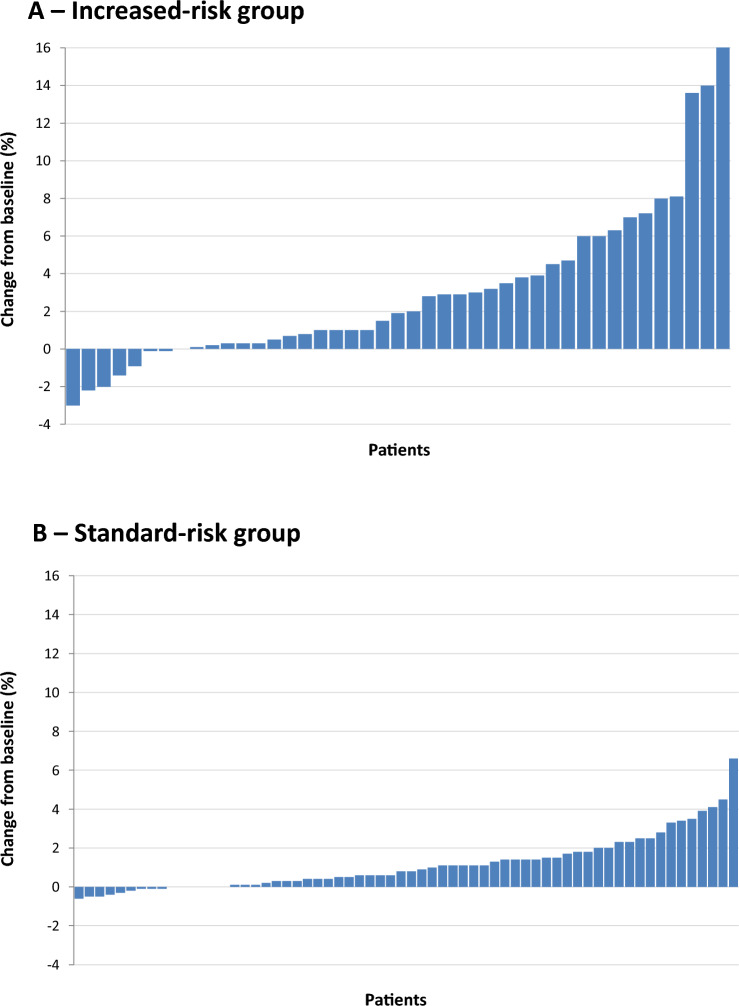
Image of individual variations in fracture risk according to the FRAX algorithm after 10 years from the start of the gluten-free diet. Percentage changes from the baseline score are reported, with patients being stratified as increased-risk group (i.e., patients with osteoporosis at the baseline dual X-ray absorptiometry or risk factors for fragility fracture—*n* = 43) or standard-risk group (*n* = 64)

A subgroup analysis was performed considering: (1) patients with osteopenia at the diagnosis and no risk factors (standard-risk group); (2) patients with osteoporosis at the index DXA or at least one risk factor (increased-risk group). In the standard-risk group (*n* = 64), the FRAX score was always < 10% at the baseline and never exceeded the 10% threshold at the end of observation (range 1.7–9.6%). Also, only one patient (1.6%) increased his score by more than 5% (Fig. 4). In the high-risk group (*n* = 43), nine (20.9%) patients started above the 10% threshold. Among them, seven remained over this threshold, and two reduced their risk; seven patients started with a < 10% risk and surpassed this threshold over time. Overall, the number of patients with a > 10% risk of major fractures was stable over time (*p* = 0.182). Moreover, ten (23.3%) patients increased their score by > 5%.

### Follow-up—incident major fragility fractures

Six major fragility fractures occurred in five (4.6%) patients during the 10-year observation time (Supplementary material). Four patients had osteoporosis and a high FRAX score at the index examination. The remaining fractured patient had low bone mineral density at the baseline DXA and began a long-term oral corticosteroid therapy for a severe case of Sjogren syndrome diagnosed 2 years after the diagnosis of CD. Among the 102 non-fractured patients, 4 had a high FRAX score at the diagnosis. Therefore, the positive predictive value, negative predictive value, sensitivity, and specificity of the 10% risk threshold were 50%, 99.0%, 80%, and 96.1%, respectively. The ROC curve analysis confirmed a good discriminative power (area under the curve = 0.826).

## Discussion

Metabolic bone disease is a relevant problem for CD patients. Still, the current guidelines for CD acknowledge that some points remain elusive and provide discordant recommendations as a consequence [28]. This study was designed to fill these gaps in knowledge and provide information to refine and, possibly, standardize these recommendations. We performed this task by conjugating a large population and an unprecedented long-term follow-up, covering more than 100,000 patient years and testing the risk estimates of the FRAX score against the actual number of fractures. Our findings bring information about the timing of the first and subsequent DXA examinations.

Regarding the timing of the first evaluation, our results confirmed a relatively high prevalence of osteoporosis at the diagnosis of CD [29], even in non-negligible proportion of younger patients. Therefore, our results support the ACG and ESsCD recommendation to perform a DXA in all adult CD patients at the diagnosis [12, 13], especially in patients > 40-year-old or with malabsorption symptoms.

However, the novel information of our study regards the follow-up. First, T-scores did not overtly deteriorate over time. This result derived from Z-scores improving, which can be explained in two ways: regularized intestinal absorption due to mucosal healing, and prompt detection and management of low bone mass. Unlike the general population with similar demographic characteristics, in fact, CD patients are continuously monitored for vitamin D and calcium levels [12–15]. Notably, the largest fluctuations in BMD were seen in patients with osteoporosis at the index measurement, while patients with osteopenia had more stable scores. Similarly, the fluctuations of the FRAX score were extremely limited in patient with osteopenia and no additional risk factors. Since the FRAX score confirmed its prognostic abilities in the setting of CD (both in this study and in a recent registry-based cohort study [27]), some implications can be drawn. In fact, our data seem to suggest that patients with osteoporosis at the index DXA should be constantly monitored, but applying the same follow-up schedule in patients with osteopenia and no risk factors could bring more limited information and benefits.

Before generalizing this hypothesis to the whole CD population, some limitations of this study should be discussed. First, the proportion of non-adherent patients was similar to that previously reported [30, 31] but still too low to analyze the relationship between adherence and densitometry modifications properly. Therefore, no inferences should be drawn from the apparent lack of correlation between non-adherence DXA parameters. These patients should continue a close monitoring regardless of their basal BMD, as only a strict GFD can avoid a persistent malabsorption [29]. Second, our study population included only a minority of post-menopausal women. Even if menopause and older age were not found to affect the BMD dynamic changes independently, we feel that future studies specifically dedicated to this population are warranted before drawing strong conclusions.

Even considering these limitations, our results still apply to a vast majority of the CD population, which usually receives a diagnosis well before the pre-menopause and adhere to the GFD. Also, our findings are corroborated by a slim proportion of the target population being excluded for incomplete data (thus making a sample bias very unlikely).

Based on these premises, a mixed approach might be proposed. DXA scans could be prescribed to all adult CD patients at the diagnosis for a comprehensive risk stratification, but with different follow-up programs in patients at high risk (which should maintain a strict 2–3 year interval) and low risk (in which a more relaxed follow-up is unlikely to lead to a loss of relevant clinical information). The FRAX score could be used at each follow-up visit to re-evaluate the timing of the next DXA. This suggestion basically consists in the application of the BSG and NICE guidelines (which suggest calculating the FRAX score without BMD information and prescribe DXA only to patients over predetermined risk thresholds) [14, 15] to the sole follow-up setting. This proposal is also in line with the recently advocated proposal of reducing the number of DXA scans in CD patients, to abate diagnosis-related time and costs [32].

In conclusion, we demonstrated that 10 years after the start of the gluten-free diet, the DXA parameters and the FRAX scores of CD patients are substantially stable compared to those found at the diagnosis. Patients without osteoporosis at the index DXA or other risk factors for fragility fracture had remarkably stable parameters and fracture risk. To reduce the time and cost of the procedures related to CD, this subgroup of patients might benefit from more relaxed follow-up without fears of losing crucial clinical information.

## Supplementary Information

Below is the link to the electronic supplementary material.

Supplementary file1 (DOCX 31 KB)

Supplementary file2 (DOCX 16 KB)

Supplementary file3 (SAV 31 KB)

## Data Availability

The authors confirm that the data supporting the findings of this study are available within the article and its supplementary materials.
