# Supplementary material for: Evolution of bone densitometry parameters and risk of fracture in coeliac disease: a 10-year perspective
Source: Intern Emerg Med. 2023 Jun 3;18(5):1405–14. doi: 10.1007/s11739-023-03307-7 (PMC10412678; doi:10.1007/s11739-023-03307-7)
Supplement: Supplementary file 2 — Supplementary file2 (DOCX 16 KB) [file 11739_2023_3307_MOESM2_ESM.docx]

**SUPPLEMENTARY TABLE:** Cases of incident major fragility fractures during the 10-year follow-up. Age, T-score, and FRAX risk refer to the parameters acquired at the diagnosis. Patient #4 experienced both spine and hip fractures during the following

| ID | Sex | Age | Risk factors | Lumbar T-score | Hip T-score | FRAX risk | Fracture | Time to fracture |
| --- | --- | --- | --- | --- | --- | --- | --- | --- |
| 1 | F | 66 | Corticosteroids | 4.2 | 3.0 | 30 | Wrist | 7y10m |
| 2 | F | 55 | None | 2.8 | 2.4 | 10 | Wrist | 3y1m |
| 3 | F | 72 | Corticosteroids* | 3.1 | 1.2 | 11 | Spine | 6y3m |
| 4 | F | 62 | Previous fractures | 3.5 | 3.0 | 17 | Spine | 2y1m |
|  |  |  |  |  |  |  | Hip | 5y4m |
| 5 | M | 36 | Corticosteroids** | 2.0 | 1.0 | 2.1 | Hip | 9y2m |

*Prescribed 5y6m after the diagnosis to treat chronic obstructive pulmonary disease.

** Prescribed 2y1m after the diagnosis to treat Sjogren syndrome.
